# Improving our understanding of future tropical cyclone intensities in the Caribbean using a high-resolution regional climate model

**DOI:** 10.1038/s41598-023-49685-y

**Published:** 2024-03-13

**Authors:** Job C. M. Dullaart, Hylke de Vries, Nadia Bloemendaal, Jeroen C. J. H. Aerts, Sanne Muis

**Affiliations:** 1 Institute for Environmental Studies (IVM), Vrije Universiteit Amsterdam, Amsterdam, The Netherlands; 2 Royal Netherlands Meteorological Institute (KNMI), De Bilt, The Netherlands; 3 Deltares, Delft, The Netherlands

**Keywords:** Climate-change impacts, Natural hazards, Physical oceanography, Atmospheric dynamics, Physical oceanography

## Abstract

The Caribbean region is prone to the strong winds and low air pressures of tropical cyclones and their corresponding storm surge that driving coastal flooding. To protect coastal communities from the impacts of tropical cyclones, it is important to understand how this impact of tropical cyclones might change towards the future. This study applies the storyline approach to show what tropical cyclones Maria (2017) and Dorian (2019) could look like in a 2 °C and 3.4 °C warmer future climate. These two possible future climates are simulated with a high-resolution regional climate model using the pseudo global warming approach. Using the climate response from these simulations we apply a Delta-quantile mapping technique to derive future changes in wind speed and mean sea level pressure. We apply this Delta technique to tropical cyclones Maria and Dorian’s observed wind and pressure fields to force a hydrodynamic model for simulating storm surge levels under historical and future climate conditions. Results show that the maximum storm surge heights of Maria and Dorian could increase by up to 0.31 m and 0.56 m, respectively. These results clearly show that future changes in storm surge heights are not negligible compared to end-of-the-century sea level rise projections, something that is sometimes overlooked in large-scale assessments of future coastal flood risk.

## Introduction

Tropical cyclones (TCs), locally known as hurricanes or typhoons, are one of the deadliest and costliest natural hazards. With eight cyclones on average per year, the Caribbean is one of the most affected regions worldwide^1^. An example of a very strong TC that made landfall in the Caribbean is TC Maria (2017) that had a disastrous impact in especially Dominica, Saint Croix, and Puerto Rico. Total damages are estimated at US$91 billion and the storm caused over 3000 direct and indirect fatalities^2^. Moreover, the damages in Dominica equalled 226% of its gross domestic product^3^. Another example is TC Dorian (2019) that made landfall in the Bahamas as a category 5 hurricane on the Saffir–Simpson scale (with maximum 1-min sustained wind speeds exceeding 82 m/s). Dorian is the strongest hurricane in modern records to strike the north-western Bahamas. TC Dorian caused at least 84 deaths and damages are estimated to be more than US$5 billion^4^. Both examples clearly demonstrate the (potentially) substantial impact of TCs in the Caribbean region and its inhabitants. Furthermore, Giardino et al.^5^ estimated that in 18 countries in the Caribbean 1.74% (453 k) and 2.96% (768 k) of the population currently lives less than 0.5 m and 1.0 m above the high tide water level, respectively. Overall, there is a clear need to assess the future impacts of TC wind speeds and storm surge in the Caribbean under climate change.

Global general circulation models (GCMs) are important tools in understanding present and future climate TC climatology^6,7^. GCMs project that the global average intensity of TCs will increase while the global average TC frequency will decrease^6,8,9^, meaning that the relative proportion of intense TCs (sustained wind speeds exceeding 58 m/s) will increase^10^. This is mainly the combined effect of a projected increase in sea-surface temperature (SST) and stronger vertical wind shear, which have an opposing influence on TC frequency and intensity. Following the maximum potential intensity theory^11^, higher SSTs mean that more energy is available for TCs to intensify. On the other hand, stronger vertical wind shear, means that winds will vary more greatly with height and limits the development and intensification process of TCs. However, there are large uncertainties associated with TC projections since GCMs exhibit large biases in the simulation of TCs in the current climate. First, the horizontal resolution of GCMs is often too coarse (100–200 km grid resolution) to simulate intense (category 4–5) TCs^12^. Higher resolution GCMs (10–50 km grid resolution) better represent the structure of category 4–5 TCs but still underestimate their wind speeds^13–16^. Second, compared to observed climate, GCMs have a stronger tendency of simulating El Niño-like climate conditions, with more vertical wind shear and consequently less TC activity in the Atlantic basin as a result^17^. Third, large SST biases exist in atmosphere–ocean coupled GCMs. A positive SST bias is the most common bias in the south-eastern tropical Atlantic, while too low SSTs are simulated along the coast of Venezuela, Brazil, and the Caribbean Sea^18^. Alternatively, SSTs can be prescribed in atmosphere-only GCMs. However, in this type of GCMs the atmosphere cannot affect SSTs which is an oversimplification of the real world^6^. The lack of SST coupling results in a surface energy imbalance which could reduce the reliability of TC intensity projections^19^.

Another type of uncertainty associated with TC risk projections originates from the fact that TCs are low-probability events, particularly at the local scale^20^. This means that robustly estimating changes in probabilities requires a large set of synthetic events^21,22^. This can be obtained from statistical modelling^23^ or from large single-model ensembles^24,25^. However, only a few of those large single-model ensembles exist, and they are generally run at coarse horizontal resolution (0.5°) which is insufficient to fully represent TCs. Moreover, probabilistic statements such as return value estimates (e.g. “Hurricane Sandy’s flood height of 2.8 m was an 1-in-400 year flood height in 2000 and is estimated to be an 1-in-90 year flood height in 2100 due to climate change”), are not always convincing in terms of the physical driving mechanisms^26^. An alternative approach is the storyline approach: instead of emphasizing probabilities originating from large climate model simulations, the storyline approach focuses on understanding the driving factors involved in changing TC intensities, and the plausibility of those factors^26,27^. This approach overcomes some of the limitations of GCMs, especially regarding the computational costs of high-resolution simulations. Moreover, a storyline approach can improve risk awareness by framing risk in an event-oriented rather than probabilistic manner^28^, more closely corresponding to how people perceive and respond to TC risk^29^. Rye and Boyd^30^ applied the storyline approach to better assess current climate TC risk, increase risk awareness, and inform decision-making. In their study, the historical path was adjusted of three major TCs that were near misses for Miami. The results revealed how much greater the impact of TCs Matthew (2016), Irma (2017), and Dorian (2019) could have been.

In this study we apply the storyline approach to show what a historical TC in a 2 °C and 3.4 °C warmer future climate could look like in the Caribbean region. We transform historical TC events Maria and Dorian to future-climate equivalents by changing the TC intensity [i.e. wind speed and mean sea level pressure (MSLP)], while assuming the TC track does not change. We refer to this change in TC intensity as the ‘Delta’ using the Δ sign. We use climate data generated with the high-resolution atmosphere-only regional climate model (RCM) RACMO2.3^31^ (referred to as RACMO from here) covering the Caribbean over the period 1979–2020. We use three RACMO experiments of which one represents the historical climate and two experiments represent a potential future climate. In our method, we track all TCs that are present in the three RACMO experiments and derive Δ’s by comparing historical and future climate from RACMO runs. These Δ’s are applied to TCs Maria (2017) and Dorian (2019) to force a hydrodynamic model for simulating storm surge levels under historical and future climate conditions.

## Results

### Validation of historical tropical cyclone characteristics

**Figure 1:**
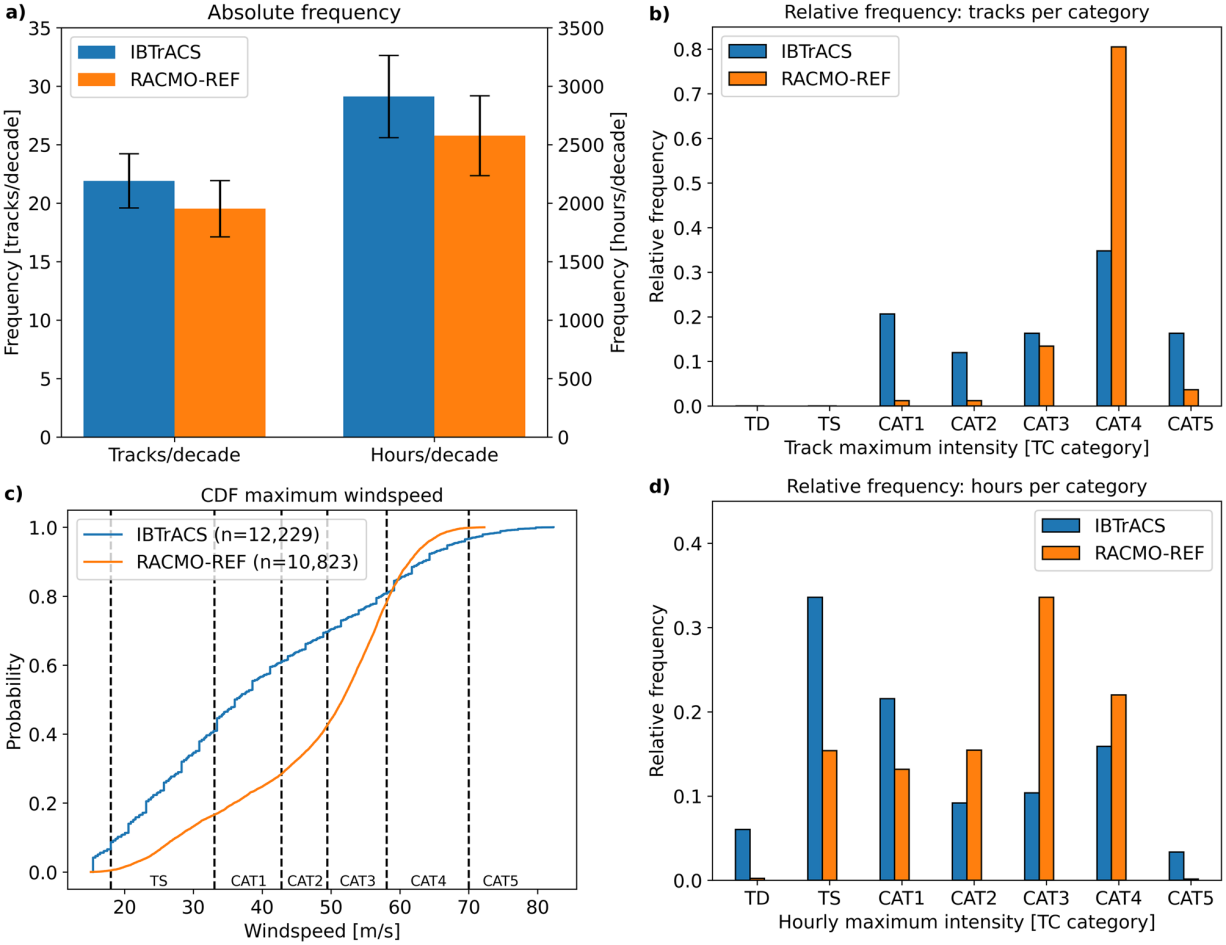
(**a**) absolute TC frequency of 1979–2020 depicted as tracks per decade (left) and hours per decade (right). Error bars represent one standard deviation that we estimate using the bootstrap method. The bootstrap method applied here consists of random sampling of the yearly TC frequencies with replacement. This procedure is repeated 10,000 times; (**b**) relative frequency of TC intensity based on TC maximum wind speeds following the Saffir-Simpson scale; (**c**) CDF of hourly TC maximum wind speeds over the period 1979-2020; (**d**) same as (**b**) but now for hourly maximum intensity.

First, we analyse how well RACMO replicates the historical TC climatology. We therefore compare various TC characteristics, such as frequency, intensity, and spatial track density, in the RACMO reference run (RACMO-REF) against observations. The RACMO-REF simulation (1979–2020) was obtained using initial conditions, and surface and lateral boundary conditions from the ERA5 reanalysis (see “Methods” section for details). Observations of historical TCs (1979–2020) are retrieved from the International Best Track Archive for Climate Stewardship (IBTrACS)^32^. Figure 1a shows that the decadal frequency of TCs in RACMO-REF is 22 TCs per decade, which is within one standard deviation from the observational record. Compared to observations, RACMO-REF has a lower number of TCs reaching at least category 1 (wind speeds exceeding 33 m/s) on the Saffir-Simpson scale, but a relatively large number of TCs reach category 3 or 4 in RACMO-REF (Fig. 1b,d). This indicates that not all TCs from the ERA5 climate reanalysis that pass the boundary of the RACMO model domain are sustained. However, when RACMO does start simulating a TC, the TC often intensifies to at least a category 3 TC. This type of behaviour is also evident from the cumulative distribution function (CDF) of hourly maximum TC wind speeds (Fig. 1c). A substantial proportion, being 53%, of the wind speed distribution of RACMO-REF falls between 50 and 65 m/s, compared to 22% for IBTrACS. By only considering TCs present in both IBTrACS and RACMO-REF, the overall alignment of the two distributions improves (Supplementary Fig. S1). This gap further reduces when considering TCs of at least category 3 intensity (Supplementary Fig. S2). This means that TCs developing within the RACMO model domain, and that are not present in IBTrACS, are generally weaker in intensity.

In total, 71% of the TCs in IBTrACS meet the criteria for rapid intensification (RI; i.e., the TC's maximum 1-min sustained wind speed increases by at least 15.4 m/s within 24 h), while in RACMO-REF 91% of the TCs undergo RI. Because RI is an important process in the development of strong TCs, it is essential that a climate model captures this process^33^. A possible explanation for the large number of TCs undergoing RI in RACMO-REF is that RACMO is an uncoupled climate model, meaning that there is no interaction between the atmosphere and the ocean. As a consequence, there is an unlimited supply of high SSTs to fuel a developing TC. When this triggers a RI process, TCs can (more) easily intensify towards category 3 or 4 intensity. At the same time, we find that the most intense wind speeds (TC category 5; ≥ 70 m/s) are still underestimated in RACMO-REF compared to IBTrACS. While the highest wind speeds in IBTrACS exceed 80 m/s, RACMO-REF’s highest wind speeds are approximately 72 m/s. This underestimation might be partly explained by the model's spatial resolution. While RACMO has a relatively high horizontal resolution (± 12 km), this is still not comparable to IBTrACS’ point-observations^34^. As a result, peak wind intensities can be averaged out against lower wind speeds within the same grid cell in RACMO.

When comparing the spatial TC track density (Supplementary Figs. S3, S4), we see that TCs in RACMO-REF are moving in a more north-westward direction compared to observations. Especially in the southern part of the model domain (between 10° and 18° N), the number of TC passages in RACMO-REF is lower than in IBTrACS. Although the TC characteristics in RACMO-REF somewhat differs from observations, we consider the performance of RACMO sufficient to look at relative changes in future TC intensity.

### Future changes in TC characteristics

**Figure 2:**
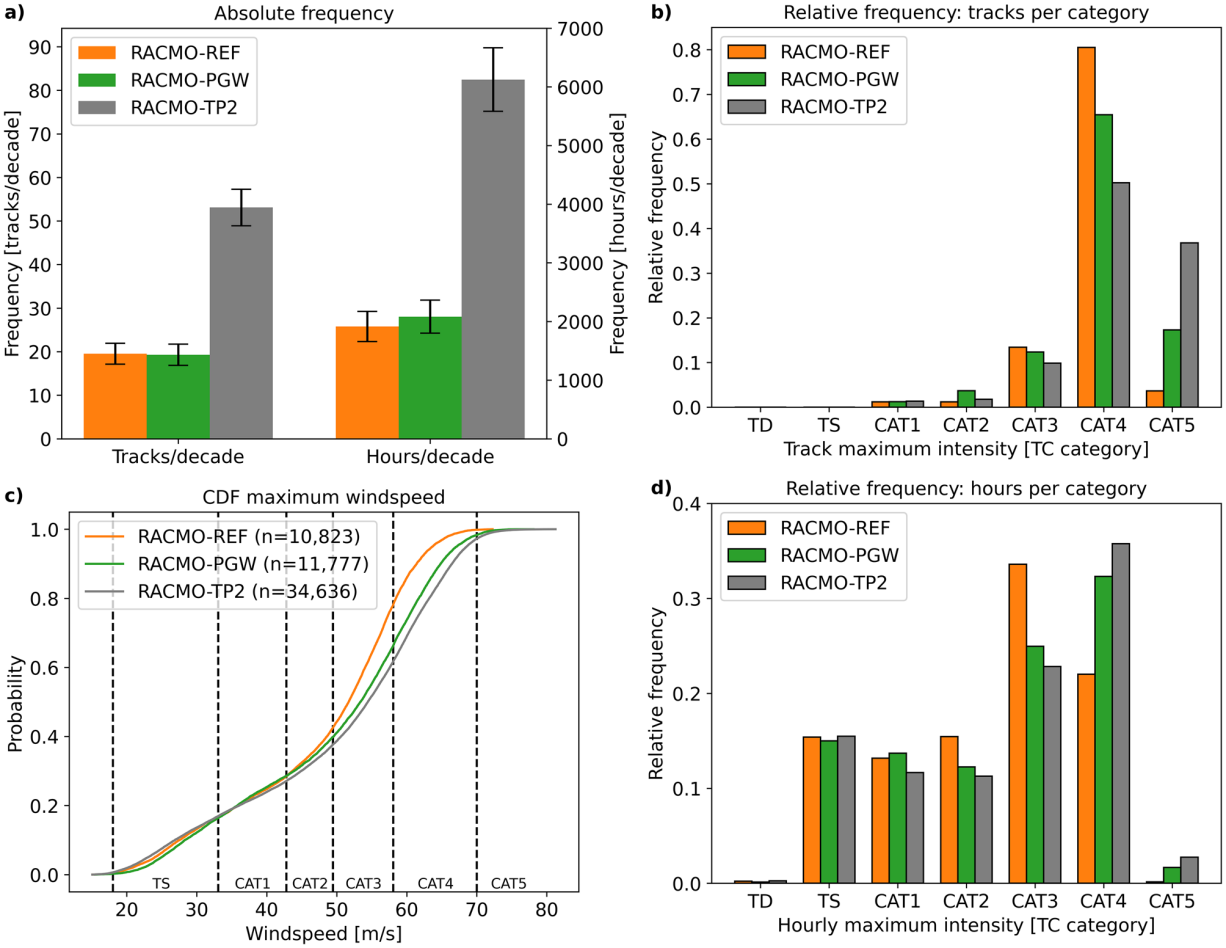
(**a**) absolute TC frequency of 1979–2020 depicted as tracks per decade (left) and hours per decade (right). Error bars represent one standard deviation that we estimate using the bootstrap method. The bootstrap method applied here consists of random sampling of the yearly TC frequencies with replacement. This procedure is repeated 10,000 times; (**b**) relative frequency of TC intensity based on TC maximum wind speeds following the Saffir-Simpson scale; (**c**) CDF of hourly TC maximum wind speeds over the period 1979–2020; (**d**) same as (**b**) but now for hourly maximum intensity.

The next step in our approach is an analysis of the differences between RACMO-REF and the two future climate experiments, RACMO-PGW and RACMO-TP2. The absolute frequency differs substantially in the two RACMO future climate experiments (Fig. 2a). These differences are caused by the assumptions about how the climate might change, underlying the two future climate RACMO simulations (see “Methods” section for a full description of the two simulations). While the absolute number of TCs reaching at least category 1 strength slightly decreases in RACMO-PGW (− 1.2%), they increase by more than a factor two and a half in RACMO-TP2 (+ 172%).

The distribution of maximum intensity along the track (Fig. 2b) shows a shift from category 4 in RACMO-REF towards category 5 in both RACMO-PGW and RACMO-TP2. This indicates that it is mostly the change in TC frequency that is different in RACMO-PGW compared to RACMO-TP2, while the TC intensity distribution is much more similar in the two future climate experiments. We observe a shift in hourly maximum TC wind speeds from category 2 and 3 towards higher intensities in RACMO-PGW and RACMO-TP2 (Fig. 2d). The CDFs of TC wind speeds in the RACMO experiments show a clear increase in maximum wind speed in both RACMO-PGW and RAMO-TP2 (Fig. 2c). Only considering those historical TCs present in all three RACMO experiments increases the difference between the CDFs for lower wind speeds (< 50 m/s), while above this threshold the increase in wind speeds from RACMO-REF to RACMO-PGW and RACMO-TP2 does not change (Supplementary Figs. S5, S6). The latter increases the certainty around the strengthening of the major hurricanes (TC category 3 and up), and is in agreement with most existing studies on future TC intensity which foresee that the more intense TCs will become stronger^6^.

Spatial density plots of TC activity in RACMO-PGW reveal a reduction of TC frequency in the southwestern part of the basin (Supplementary Figs. S7, S8). Most likely, this is caused by the different large-scale dynamics in the RACMO-PGW experiment, leading to stronger wind shear conditions which are less favourable for TC development and intensification in this area. The spatial density plot of RACMO-TP2 shows a pattern that is more comparable to RACMO-REF, with predominantly the absolute TC frequency changing in this experiment due to the modelled 2 °C temperature increase.

### Delta (Δ): tropical cyclone intensity change and application

**Figure 3:**
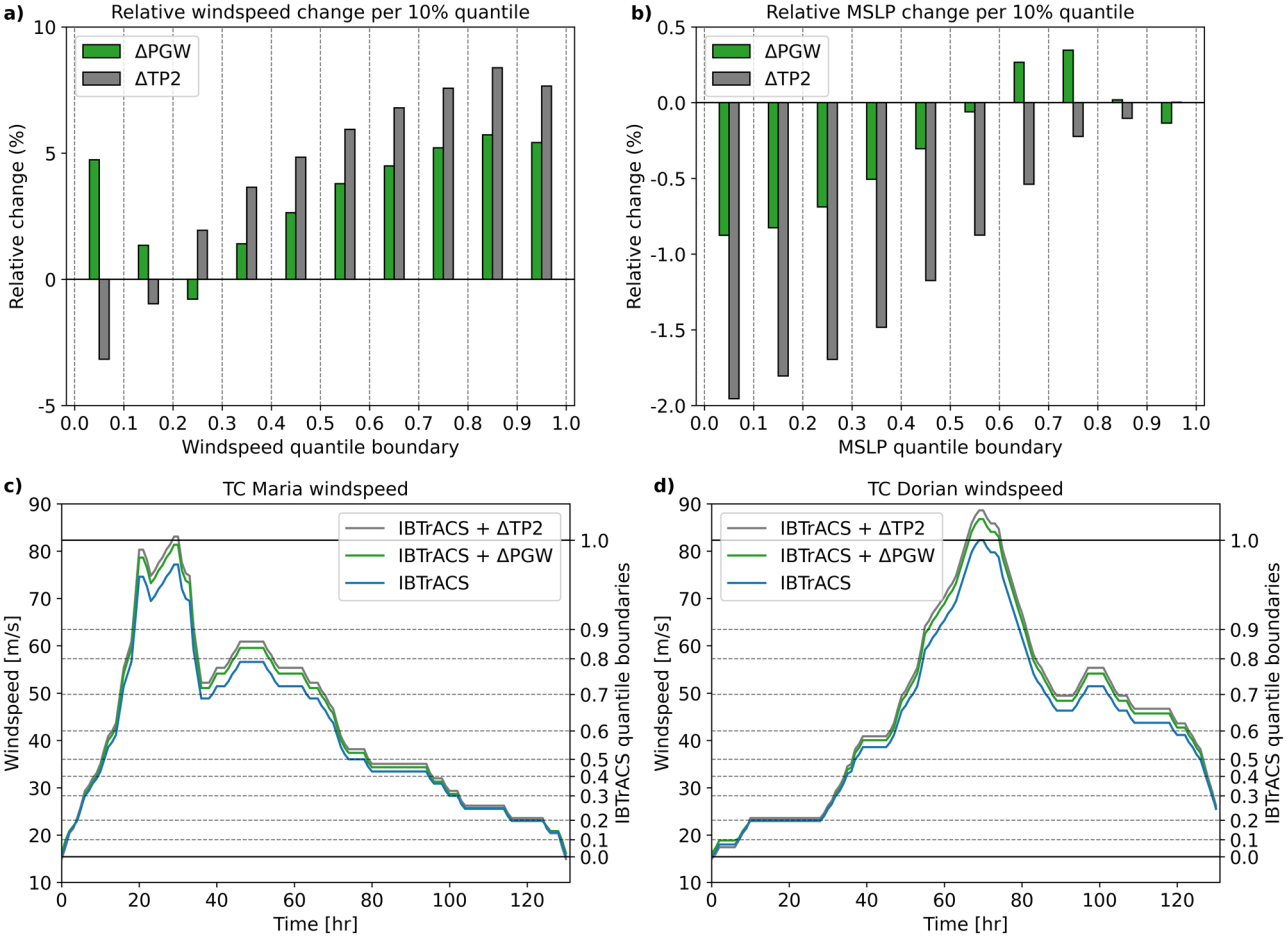
(**a**) bar plot showing the change in average hourly maximum wind speed per 10% quantile for TCs; (**b**) same as panel (**a**) but for MSLP; (**c**) and (**d**) show the wind speed of TCs Maria and Dorian over time with the blue line indicating observed wind speeds from IBTrACS, and the green and grey line indicating the adjusted wind speeds based on RACMO-PGW, and RACMO-TP2, respectively. The secondary y-axis indicates the 10% quantile boundaries of the hourly maximum TC wind speeds (1979–2020) from IBTrACS.

To demonstrate the Δ method using the storyline approach, we simulate TCs Maria and Dorian’s storm surges using the historical TC track data and the adjusted tracks based on ΔPGW and ΔTP2 as forcing for the Global Tide and Surge Model (GTSM). The first method that we apply to compute a Δ representing the change in TC intensity is taking the relative change in the average track maximum wind speed. When taking all storm events into account reaching at least tropical storm strength (wind speed > 18 m/s), the average track maximum wind speed increases by 3.88% in RACMO-PGW and 7.13% in RACMO-TP2, compared to RACMO-REF. Knutson et al.^6^ presents a meta-analysis based on 52 studies that also use this approach and reported a mean projected change in TC maximum wind speed of + 3% (10th and 90th percentiles are + 0.5% and + 6.5%, respectively) for the North Atlantic. This means that the ΔPGW value that we find fall within the 10th–90th percentile of these 52 studies, while the ΔTP2 value is slightly above the 90th percentile. However, a downside of this method is that the Δ value only represents the change in average TC maximum intensity. As shown in "Future changes in TC characteristics", the shape of the TC wind speed distribution changes as well, advocating for the use of an intensity-dependent Δ method. Therefore, we also design a Δ method that makes use of quantile mapping (see “Methods” section). The change in average wind speed per 10% quantile shows a similar pattern in RACMO-PGW and RACMO-TP2 compared to RACMO-REF (Fig. 3a). The negative change at the lower quantiles shown by RACMO-TP2 might be caused by the large amount of TCs that develop within the RACMO model domain in this experiment. In addition, we compute the Δ-values based on TCs that are present in all RACMO simulations (Supplementary Figs. S9, S10) which removes most of the TCs that develop within the model domain. This removes the large negative change at the lower wind speed quantiles. Above the 30% quantile we find an increase in wind speeds for both RACMO-PGW and RACMO-TP2, indicating that the relative change is more robust at the higher quantiles. For MSLP, the ΔPGW and ΔTP2 show a similar pattern over the quantiles as for wind speed, with the right-tail MSLP values around zero, and the left-tail MSLP values decreasing even further (i.e., the strongest TCs are intensifying even further; Fig. 3b). However, one difference compared to the wind speed is that for MSLP, ΔTP2 is much larger (i.e. stronger pressure drop) for the most extreme 10% quantile (between 0 and 0.1) compared to ΔPGW. A potential explanation for this is that in RACMO-TP2 the TCs tend to grow ‘bigger’ (i.e., a larger area with a significant pressure decrease) compared to RACMO-PGW, which allows the MSLP to decrease further in RACMO-TP2. The radial pressure gradients however (responsible in part for the wind speed), appear to increase less.

**Figure 4:**
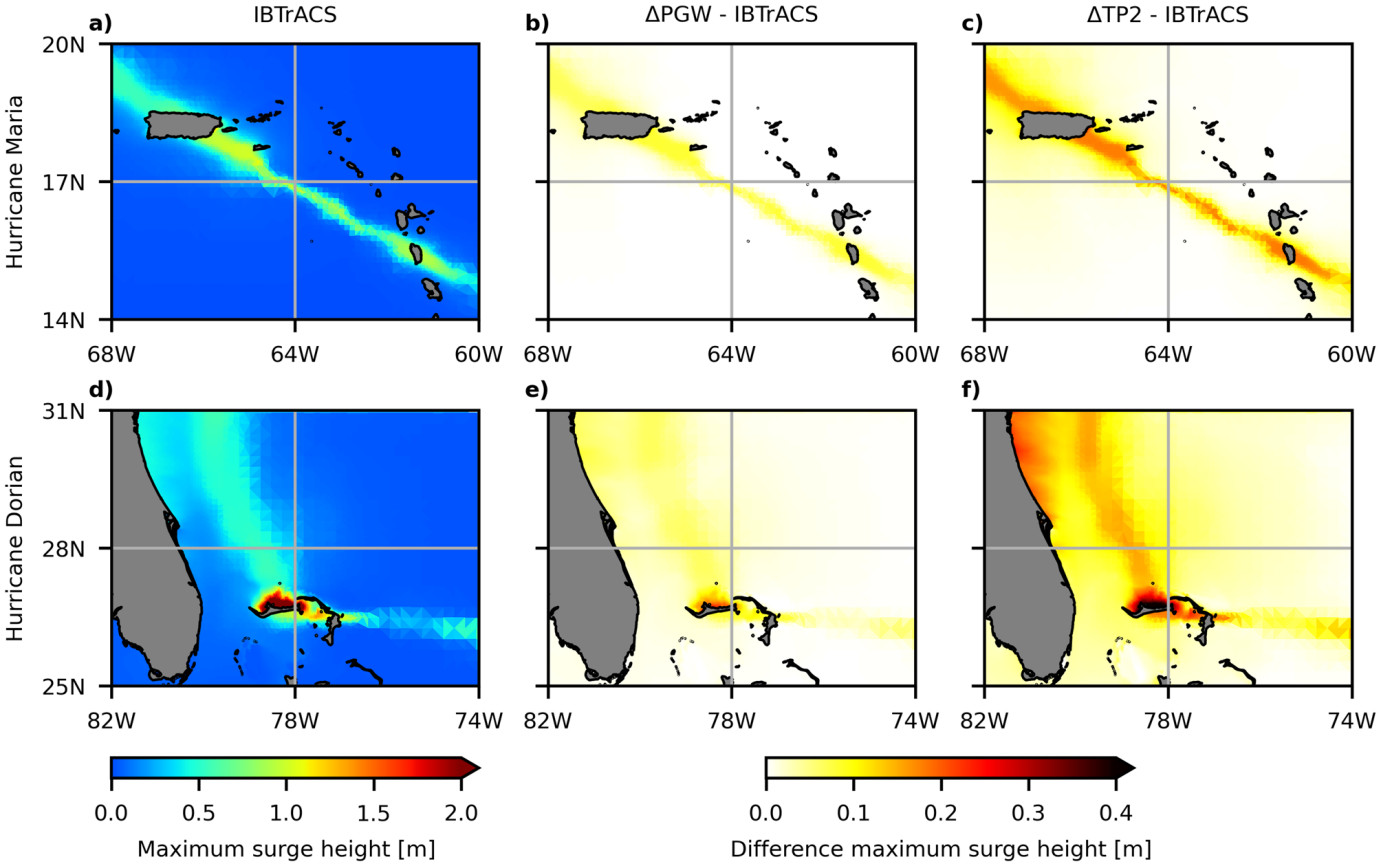
(**a**) maximum surge height of Hurricane Maria using the observed TC track as forcing; (**b**) difference in maximum surge height using ΔPGW, calculated as ΔPGW minus IBTrACS; (**c**) same as (**b**) but for ΔTP2; (**d**–**f**) same as (**a**–**c**) but for Hurricane Dorian.

Subsequently, we apply the Δ method to TCs Maria and Dorian (Fig. 3c,d). While Maria’s highest wind speed remains within the observed range of IBTrACS, Dorian’s maximum wind speed exceeds the highest observed wind speed that is present in the IBTrACS dataset (see upper horizontal black line (Fig. 3d)). Figure 4 shows the maximum storm surge height. Note that we adjusted both the wind speed and MSLP values using the Δ values shown in Fig. 3a,b. The maximum simulated surge height for TC Dorian is much higher (3.9 m) compared to TC Maria (1.5 m). This is mainly due to the coastal morphology of the respective landfall locations: Dorian made landfall in the Bahamas, an archipelago located on a shallow coastal shelf. Maria, on the other hand, made landfall in Puerto Rico, an island with a steeper coastal shelf. As storm surge heights tend to be further amplified in shallow coastal areas^35^, this can be a possible explanation for Dorian’s higher storm surge despite the TC intensity being roughly the same amongst the two TCs. For TC Maria, the maximum surge height increases in the future climate simulations by 0.14 m (ΔPGW) and 0.31 m (ΔTP2). The increase in maximum surge height is larger for TC Dorian with 0.29 m (ΔPGW) and 0.56 m (ΔTP2). It is mainly the lower MSLP (Supplementary Fig. S11) that is responsible for the larger increase in surge height in the ΔTP2 simulation, compared to ΔPGW. Differences in maximum wind speed between the ΔPGW and ΔTP2 simulation are smaller (Supplementary Fig. S12). Our results indicate the potentially increased impacts of TCs that could occur in a future warmer climate. Although the possible increase in TC surge height is smaller than the projected end-of-the-century sea level rise (SLR) (0.5–1.0 m, depending on the Shared Socioeconomic Pathway (SSP) scenario)^36^, the increase is not negligible and should be accounted for. In addition, the results demonstrate that the impact of climate change on future TC intensity and storm surge height can be presented in an understandable way using the storyline approach. Steps that would be required for this are: (1) including other water level components such as waves, swell, and tides; (2) inundation modelling; and (3) assess the impacts in terms of flood and wind damage, and the number of people exposed.

## Discussion

In this study we developed a novel Δ method and used the storyline approach to gain insight in future changes in TC intensity and storm surge height due to climate change in the Caribbean. The storyline approach enabled us to overcome the computational limitations of using a probabilistic method based on GCMs. Instead, our method is computationally efficient and enhances risk awareness by framing risk in an event-oriented rather than probabilistic manner. The storyline approach uses historic and future RCM simulations to compute a Δ value representing projected future changes in TC intensity based on quantile-mapping. Applying these Δs to TC Maria and TC Dorian showed that, depending on how the climate will change, maximum storm surge heights could increase by up to 0.31 m and 0.56 m, respectively. These results clearly show that future changes in storm surge heights are not negligible compared to end-of-the-century sea-level rise projections, something that is sometimes overlooked in large-scale assessments of future coastal flood risk.

Several aspects of our methodology could be improved. The first aspect relates to limitations linked to the use of RACMO experiments as input. RACMO is an atmosphere-only RCM, meaning that SSTs are prescribed with no atmosphere–ocean interaction. As a result, TCs may intensify too rapidly^37,38^. In reality, the TC-induced ocean cooling effect (i.e. mixing of the surface water with colder subsurface water) reduces SSTs, constraining a TC’s intensification^18,19^. This shortcoming could be addressed by using an atmosphere–ocean coupled climate model, although, these models are computationally much more expensive, making them less easily applicable for risk assessment frameworks as presented in this study^39–41^. Furthermore, such coupled models have shown to also have their shortcomings such as severe SST biases in the tropical Atlantic ^18,42,43^. Another shortcoming of RACMO is the relatively short length of the climate experiments (42 years). Because TCs are relatively rare events^20^, the limited record length means that the number of TCs is rather small to accurately assess future changes in TC intensity in the Caribbean. To overcome this, larger climate model simulations (e.g., longer simulation periods or using single-model ensembles) could be carried out. Last, the spatial resolution of RACMO is relatively high for a climate model (12 × 12 km), but it still falls short of the scale and nature of point data as is collected, for example, on meteorological stations and ocean buoys^39^. This might explain why the number of TCs reaching category 5 intensity in RACMO-REF is lower compared to observations (IBTrACS), as climate data generated with RACMO is more homogenous in space compared to point observations^44^.

Second, large uncertainty exists around the impact of climate change on the El Niño southern oscillation (ENSO) which has a large influence on TC activity in the Atlantic^45–48^. Global circulation models and historical observations of ENSO are currently not in agreement. Climate trends derived from observations are hinting for a La Niña climate with less wind shear in the Caribbean region and thus an increase in TC activity in the area^49^. GCMs on the other hand, have a stronger tendency of moving towards an El Niño climate, with more vertical windshear and consequently less TC activity in the Atlantic basin^17^. This makes it interesting to look at the differences in TC activity in RACMO-PGW and RACMO-TP2. Roughly speaking, RACMO-PGW is more in agreement with El Niño conditions, while RACMO-TP2 better replicates La Niña conditions. Comparing both model simulations in terms of TC frequency is not possible as the Main Development Region (MDR)^50^, the dominant birth chamber for TCs in the Atlantic Ocean, is located outside of RACMO’s model domain. One way to solve this would be to increase the model domain such that it also includes the MDR. Alternatively, forcing from GCMs could be used. Despite this, the current model setup does support analysis of TC intensity within the model domain. While both model simulations reveal an increase in TC intensity, the increase seems to be larger in RACMO-TP2. This is in agreement with existing studies on the influence of ENSO on TC intensity in the Atlantic basin^51,52^.

Third, when computing the Δ values per 10% quantile we take all tracked TCs into account. However, even though ERA5 data is prescribed on the RACMO's model’s boundary, the number of TCs strongly differs between the three RACMO simulations. Especially in the RACMO-TP2 experiment many TCs develop within the model domain, resulting in a larger share of relatively low TC hourly maximum intensities. The Δ values representing the lower quantile wind speed changes (lower 30% quantiles) are therefore sometimes negative, up to − 3%. We found that only taking TCs into account that are present in all RACMO experiments changes the sign of these Δ values from negative to positive. Because a higher amount of events will reduce the uncertainty, we decided to use all TCs for computing the Δ values. In addition, a large relative decrease of lower TC wind speeds remains small in absolute terms (e.g. minus 0.6 m/s at a wind speed of 20 m/s). However, despite these uncertainties, the Δ values representing the higher quantiles (upper 60% quantiles) show a robust increase, independent from whether all tracked TCs or only the matched TCs are taken into account.

Fourth, our application of the storyline approach to assess future changes in storm surge heights could be improved. Aspects that could be included are waves and swell^53,54^, tides^55^, precipitation^56,57^, and the impact of climate change on these factors, as well as sea-level rise^36^. In addition, the assessment could be expanded by performing a complete coastal flood risk assessment that includes the modelling of coastal flooding^58^. Our storyline approach could be used for example to inform inhabitants of Small Island Developing States^59^ about their current and future TC flood risk in an understandable way. This could improve risk awareness and TC readiness, eventually resulting in better protection from TC impacts.

To conclude, this study provides a novel Δ method to show how climate change might impact TC intensity and related storm surge heights. We developed a method to compute a Δ that represents the possible change in TC intensity under climate change. In contrast to existing methods, our method is intensity dependent, meaning that the intensity at a certain timestep of the historical TC track is accounted for. In addition, presenting the impact of climate change on TCs and storm surge in the Caribbean using the event-based storyline approach makes findings easy interpretable for locals and non-specialists since they are aware of the historic events that are placed into the future. Last, our results show that, contrary to common assumptions made in flood risk assessments, future increases in storm surge heights are not negligible with respect to SLR projections.

## Methods

### General approach

To generate possible future climate versions of TCs Maria (2017) and Dorian’s (2019) intensities and corresponding storm surges we follow five main steps. First, we track all TCs that are present in the three RACMO climate experiments. Second, we compute a number of statistical indicators to assess how well RACMO replicates the observed TC climatology (1979–2020). Third, we analyse how the TC intensity changes in the two RACMO future climate experiments that follow different assumptions about how the climate will change. Fourth, we translate the TC changes to different change factors, a so-called "Delta (Δ)", using quantile mapping. This Δ can for example replicate the change in average TC lifetime maximum wind speed or minimum MSLP between the historical and future climate experiments. Subsequently, this Δ can be used to adjust the hourly maximum wind speed and MSLP values of a historical TC track such that it replicates the effects of climate change on TC intensity. Fifth, we apply the different Δs to the observed track of TCs Maria and Dorian to compute a future version of the two events and force a hydrodynamic model with the historical and the future climate TC to simulate storm surge levels.

### Description RACMO climate model experiments

**Figure 5:**
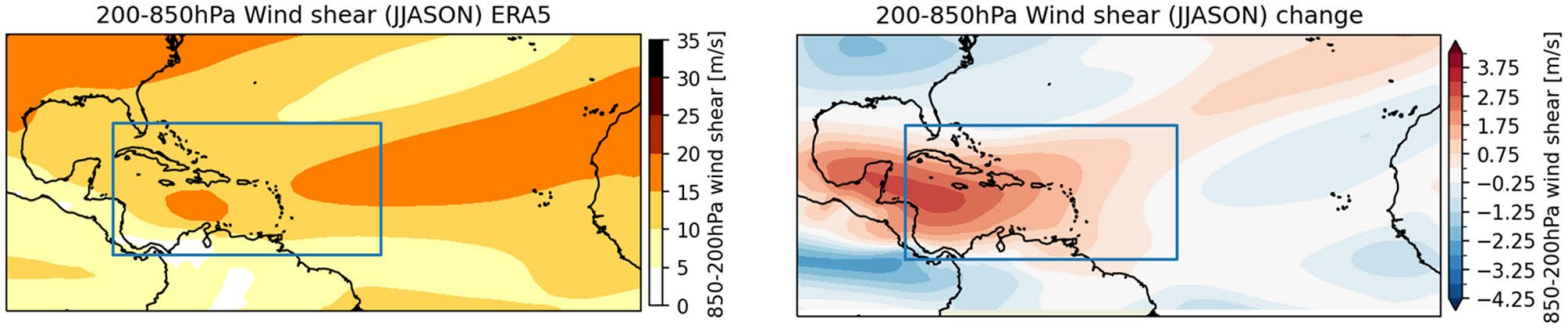
ERA5 vertical wind shear average over the months June–November and years 1970–2020 (left) and the projected change derived (right) from CMIP5 under climate change scenario RCP8.5 and an increase in global average temperature of 3 °C. The blue box denotes the approximate RACMO simulation domain.

We use of a set of present-day and future RCM simulations obtained with the regional climate model RACMO2.3^31^, developed as part of the Horizon 2020 European Climate Prediction system (EUCP) project^60,61^. RACMO is a hydrostatic RCM that operates at a horizontal resolution of 12 × 12 km, and has 40 layers in the vertical. External forcings and greenhouse gases are taken from the fifth phase of the Coupled Model Intercomparison Project (CMIP5)^62^. The approximate model boundaries are shown in Fig. 5. SSTs are prescribed and not affected by the passage of a TC. In reality, the TC-induced ocean cooling effect reduces SSTs, constraining a TC’s intensification. Because of boundary uncertainty, we removed all RACMO output data within 3° of the climate model boundary (± 300 km at the model’s domain average latitude). Boundary uncertainty is due to the fact that RACMO has been run under the constraint of the ERA5 global climate reanalysis. As a result, TCs need some time after entering the model domain to adjust to the new climate conditions. In addition, TCs are sometimes not able to leave the model domain when the TC is blocked by the prescribed boundary conditions. Because natural variability in TC activity is large, long simulations are required to reliably estimate future changes in TCs. Since high-resolution simulations are computationally expensive, the experimental design of EUCP uses the Pseudo Global Warming (PGW) approach^63^. This means that the future simulations are produced by adding a slowly (i.e. monthly) varying ‘delta-change’ signal to the original high-frequent ERA5 lateral boundary data. Within the simulation domain the model is free to adapt to new conditions. The PGW experiment consists of a reference and two future simulations, which we here briefly explain.

1. RACMO-REF: A reference simulation is generated using the ERA5 reanalysis data^64^ at lateral boundaries and as initial conditions (a 3-h update scheme was used). The reference simulation covers the years 1979–2020 and the months June to November. Each year a new simulation was started at the first of June, the approximate start of the North-Atlantic TC season.
2. RACMO-TP2: The simplest future PGW disturbance was to add a uniform 2K warming to boundaries, SST and soil moisture. In the vertical, the moisture field is also adjusted such that the relative humidity is kept constant. Without this modification of the moisture profile, the increase in temperature would lead to too strong reductions in relative humidity. This simulation is referred to as the RACMO-TP2 simulation. Important to note is that in RACMO-TP2 there is no change at the boundaries of the large-scale circulation (winds), nor of the vertical stratification.
3. RACMO-PGW: The other future PGW simulation adds a 3D future delta field derived from CMIP5 ensemble projections and the RCP8.5 scenario. These delta fields (derived for temperature, pressure, zonal and meridional wind and moisture) comprise of the end-of-century (2071–2100, RCP8.5) minus present-day (1976–2005) ensemble mean differences derived from a subset of 19 CMIP5 GCMs^65^. The warming level for these simulations, to which we refer as RACMO-PGW, is 3.4 K. The RACMO-PGW simulations, therefore, have a considerably stronger warming level than the RACMO-TP2 runs but augment them with the consistent changes in large-scale circulation, vertical stability, and wind shear. The average historical wind shear from ERA5 and projected change based on CMIP5 are shown in Fig. 5.

### Tropical cyclone tracking

To capture the TC statistics in each of the three RACMO climate model simulations, we apply a TC tracking algorithm. This algorithm consists of six main steps. First, we interpolate the 1-h RACMO data from a rotated polar oriented grid to a regular grid with a spatial resolution of 0.1**°** × 0.1**°** (± 11 km at the equator) using bicubic interpolation. The spatial resolution of 0.1**°** × 0.1**°** is selected because this is similar to RACMO’s original resolution of 12 × 12 km. Second, following Gutmann et al.^66^, we start tracking a point (i.e., a local minimum of MSLP) when the MSLP falls 17 hPa below the yearly maximum MSLP and the maximum wind speed within a 300 km radius of this point exceeds 15 m/s. Third, we select those points of which the MSLP is a local minimum in the 2D MSLP field at a certain timestep. This reduces the number of points that are tracked and improves the efficiency. We do not put an upper limit on the number of local minima found because multiple TCs can be active simultaneously within the RACMO model domain. Fourth, we create a track by combining points if they are detected in two adjacent time steps and are less than 300 km apart. Fifth, following Gutmann et al.^66^, we filter the TCs by only keeping a track in the analyses fulfilling the following requirements: (1) the wind speed exceeds 25 m/s over its lifetime; (2) the MSLP drops 27 hPa below the yearly maximum over its lifetime; and (3) the track duration is at least 72 h.

### Analysis of tropical cyclone characteristics

Our analysis of the TC tracks consists of two steps. As a first step, we assess the RACMO model performance by comparing TCs from RACMO-REF against TCs from the International Best Track Archive for Climate Stewardship (IBTrACS)^32^. IBTrACS contains a complete record of TC observations since the 1980s. We compute and compare: (1) absolute frequency of TC hours and tracks; (2) relative frequency of TC hours and tracks per TC category following the Saffir-Simpson scale; (3) cumulative distribution function (CDF) of hourly maximum TC wind speeds; (4) TC track-point density aggregated to a 1 degree resolution; (5) CDF of hourly maximum TC wind speeds that are present in RACMO-REF and IBTrACS; (6) number of TCs that meet the criteria for rapid intensification (RI; i.e. an increase in maximum sustained winds of at least 15.4 m/s in a 24-h period). Note that (4) and (5) are part of the *supplementary materials*.

In the second step, we compute the same indicators for the RACMO-TP2 and RACMO-PGW experiments. This is then compared against the RACMO-REF experiment to assess future changes.

### TC intensity change Delta (Δ)

Based on the RACMO-PGW and RACMO-TP2 climate experiments compared to RACMO-REF, we compute a Δ that represents the change in TC intensity resulting from climate change. Different methods exist for computing a Δ. Because of the used PGW approach, meaning that ERA5 reanalysis data is prescribed at RACMO's boundary, TC tracks are present in all three RACMO experiments at the model’s boundary where the TCs enter the model domain. Therefore, the most straightforward method to compute a Δ, would be to compare a certain TC in all three RACMO experiments directly. However, TCs are a stochastic process with a large degree of freedom in the model, meaning that a climate model will never simulate the exact same track but that there is a lot of randomness. In addition, RACMO simulates changes in the large-scale atmospheric conditions. This means that simulated TC tracks can start to differ in the three RACMO experiments after they have entered the model domain due to differences in steering flows and locations of high- and low-pressure areas. This may affect TC intensification, as TCs could potentially be advected over regions that are more (less) favourable for intensification. Moreover, due to differences in local bathymetry and coastline geometry, a small shift in a TC’s path can result in a completely different storm surge and flood extent^67^. Therefore, applying the event-based storyline approach directly on RACMO climate model output is not feasible.

Following previous studies (e.g., Shepherd et al.^26^, Hegdahl et al.^28^, Sillman et al.^68^, and Pitman et al.^69^), another method to obtain a Δ is by computing the relative change in TC track maximum intensity in the future experiment compared to the reference experiment. Knutson et al.^6^ present a meta-analysis based on 52 studies that use this approach and reported a mean projected change in TC maximum intensity (10-m surface wind speed) of + 3% (10th and 90th percentiles are + 1% and + 7%, respectively) for the North Atlantic. Note that these values include tropical storm systems (wind speeds between 18 and 33 m/s). Here, we compute a Δ using the same method, and compare results against Knutson et al.^6^. A downside of this method is that the Δ value only represents the change in average TC maximum intensity. Applying such TC maximum intensity Δ to the complete track of a historical TC that reached category 5 on the Saffir–Simpsons scale might be wrong. This is because the intensity change could be larger (or smaller) at lower TC intensities (e.g. while a TC is at category 2 intensity). This could be improved by computing a TC intensity dependent Δ, such that the Δ depends on the original intensity of the historical TC at the respective timestep.

To this end, we develop a new method to compute a TC intensity dependent Δ based on quantile mapping. Quantile mapping is a commonly applied bias correction method to correct systematic distributional biases in outputs of climate models^70^. The method that we apply here consists of four main steps. First, we compute the average of the 10% quantiles of hourly maximum TC wind speeds from RACMO-REF to both RACMO-PGW and to RACMO-TP2. This way, we have one Δ value per 10% quantile and thus a total of ten Δ values per future climate RACMO experiment. To be able to apply these Δ values to the wind speeds of a historical TC track we need to evaluate to which quantile (within the observed TC dataset IBTrACS) each of the wind speed values belongs. As such, the third step is to calculate the 10% quantile boundaries (wind speed in m/s) of the IBTrACS dataset with observed tracks of TCs (1979–2020), and categorize the wind speeds of the historical track based on these quantiles. Fourth and last, we multiply each wind speed value of the historical TC track with the corresponding Δ value. For example, if we have computed a ΔPGW value of + 10% for the 60–70% quantile of IBTrACS (observed wind speeds between 45 and 52 m/s), and the wind speed is 50 m/s at a certain timestep of a historical TC, we increase this wind speed by 10% such that is becomes 55 m/s (see “Results” section for a visualization). This procedure is repeated for all hourly maximum wind speeds of the historical TC. Note that we explain this method here using wind speeds as possible application, but this method can also be applied to other TC parameters such as MLSP.

### Applying the Δ to two historical events

In this study, we apply the TC intensity dependent Δ method to TC Maria and TC Dorian and assess what future versions of these storms could look like. Maria (2017) made landfall in Dominica and Puerto Rico, and Dorian (2019) made landfall at peak intensity in The Bahamas. Both TCs reached category 5 on the Saffir-Simpson scale, making them interesting case studies. For each TC we have three versions: (1) IBTrACS; (2) IBTrACS + ΔPGW; (3) IBTrACS + ΔTP2. TC track data is translated into spatial fields of wind speed and MSLP by using Holland’s parametric wind model^71^, see Dullaart et al.^72^ for details. Subsequently, storm surges of TC’s Maria and Dorian are simulated with the fourth generation Global Tide and Surge Model (GTSMv4.1)^73,74^. The GTSMv4.1 is a global depth-averaged hydrodynamic model based on the Delft3D Flexible Mesh software^75^. The resolution varies between 25 km in deeper parts of the ocean and 2.5 km along the coast (1.25 km for Europe). Storm surges can be simulated by forcing GTSMv3.0 with wind and pressure fields. The stress exerted by the wind on the ocean is parameterized by multiplying the quadratic 10-m winds with the atmospheric density and a user-defined wind drag coefficient (Cd), which depends on the surface roughness^76,77^. Here, we use the same model setup as described in Ref.^72^.

### Supplementary Information

Supplementary Information.

## Data Availability

The Delta (Δ) method developed in this study and TC tracking scripts are available from Github at https://github.com/jobdullaart/TC_Delta_method.
