# Supplementary material for: Improving our understanding of future tropical cyclone intensities in the Caribbean using a high-resolution regional climate model
Source: Sci Rep. 2024 Mar 13;14:6108. doi: 10.1038/s41598-023-49685-y (PMC10937637; doi:10.1038/s41598-023-49685-y)
Supplement: Supplementary file 1 — Supplementary Information. [file 41598_2023_49685_MOESM1_ESM.pdf]

Supplementary information

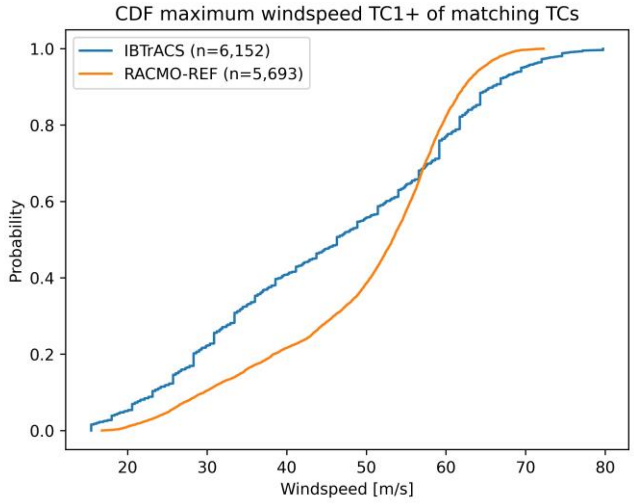

Supplementary Figure S1: CDF of hourly TC maximum wind speeds of the period 1979-2020 with data from IBTrACS shown in blue and RACMO-REF in orange. Hourly maximum wind speeds of TCs that reach at least category 1 on the Saffir-Simpson scale are shown. In addition, values are only shown if TCs are present in both IBTrACS and RACMO-REF. The threshold for matching TCs is that a TC in RACMO-REF is within 300 km from a TC in IBTrACS at a single timestep.

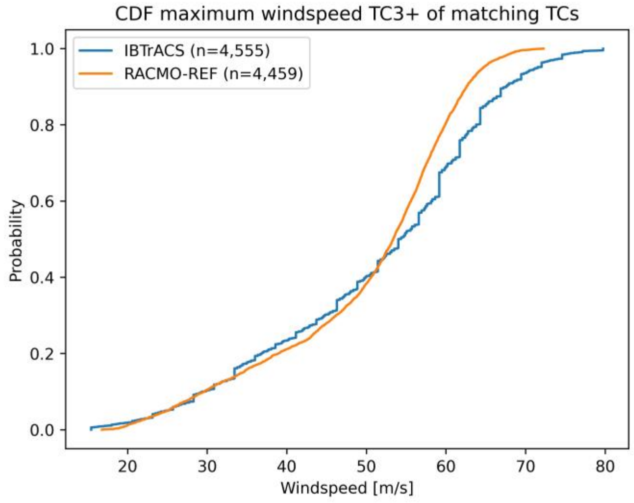

Supplementary Figure S2: CDF of hourly TC maximum wind speeds of the period 1979-2020 with data from IBTrACS shown in blue and RACMO-REF in orange. Hourly maximum wind speeds of TCs that reach at least category 3 on the Saffir-Simpson scale are shown. In addition, values are only shown if TCs are present in both IBTrACS and RACMO-REF. The threshold for matching TCs is that a TC in RACMO-REF is within 300 km from a TC in IBTrACS at a single timestep.

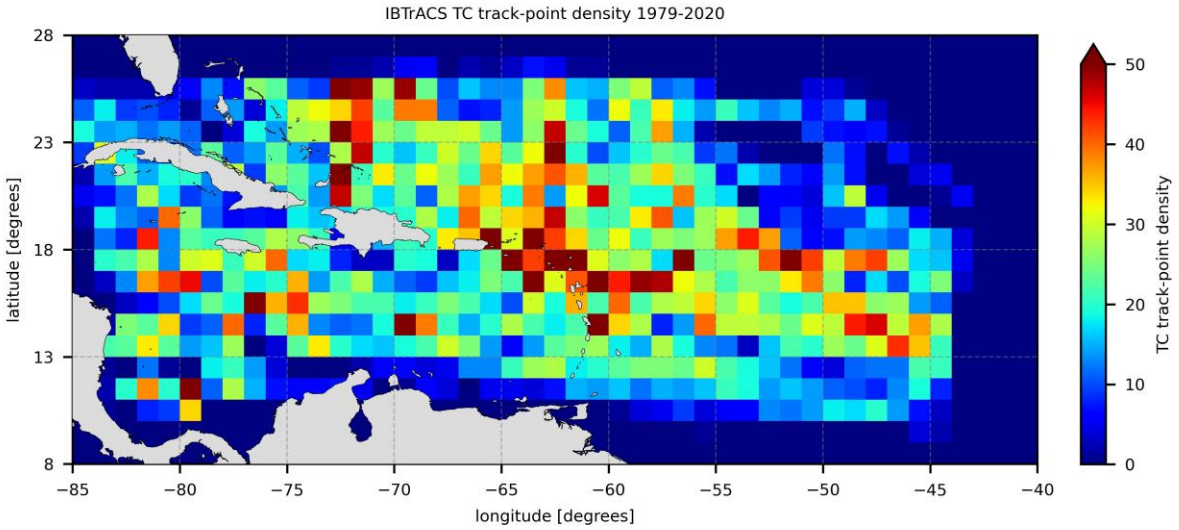

Supplementary Figure S3: Observed TC track-point density (IBTrACS) within the RACMO model domain. Units are cumulative 1-hourly storm position frequency per 1° x 1° gridbox for the period 1979-2020.

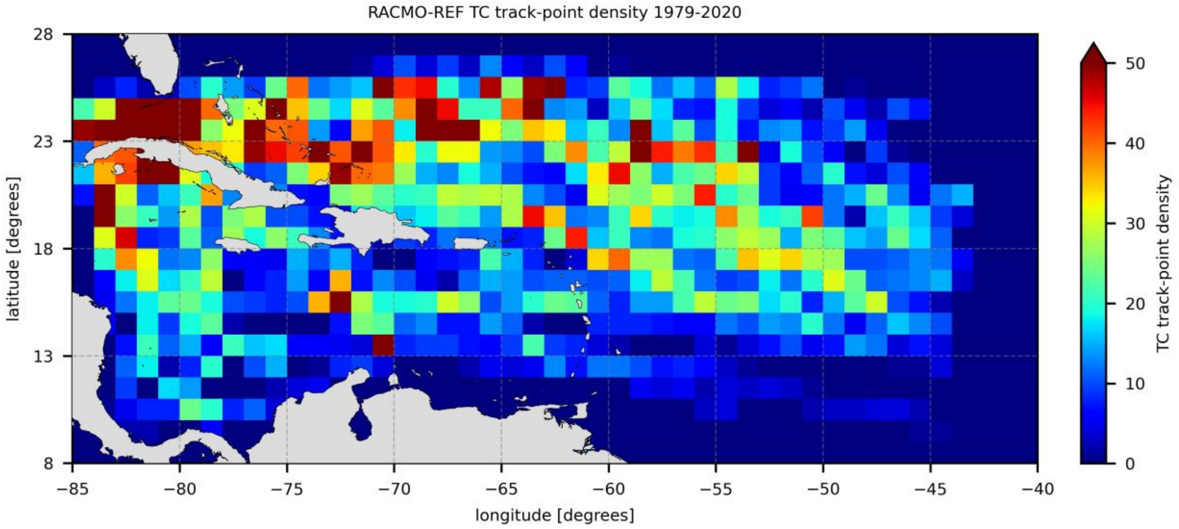

Supplementary Figure S4: Simulated TC track-point density (RACMO-REF) within the RACMO model domain. Units are cumulative 1-hourly storm position frequency per 1° x 1° gridbox for the period 1979-2020.

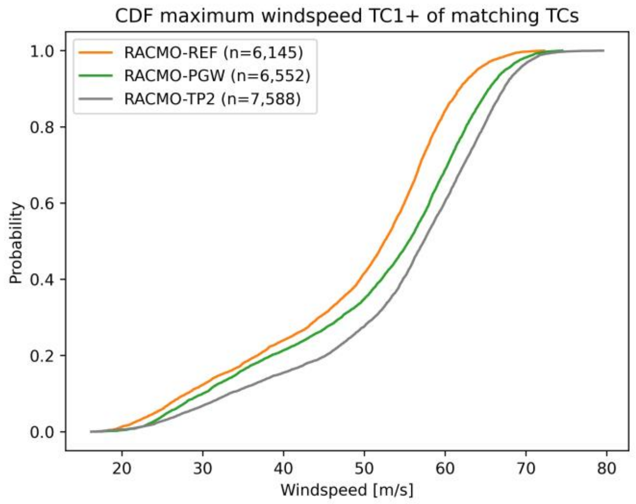

Supplementary Figure S5: CDF of hourly TC maximum wind speeds of the period 1979-2020 with data from RACMO-REF (orange), RACMO-PGW (green), and RACMO-TP2 (grey). Hourly maximum wind speeds of TCs that reach at least category 1 on the Saffir-Simpson scale are shown. In addition, values are only shown if TCs are present in all three RACMO experiments. The threshold for matching TCs is that a TC in RACMO-REF is within 300 km from a TC in both future climate RACMO experiments.

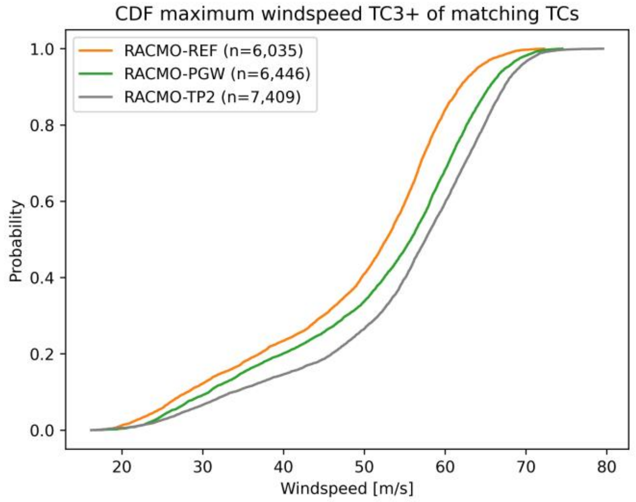

Supplementary Figure S6: CDF of hourly TC maximum wind speeds of the period 1979-2020 with data from RACMO-REF (orange), RACMO-PGW (green), and RACMO-TP2 (grey). Hourly maximum wind speeds of TCs that reach at least category 3 on the Saffir-Simpson scale are shown. In addition, values are only shown if TCs are present in all three RACMO experiments. The threshold for matching TCs is that a TC in RACMO-REF is within 300 km from a TC in both future climate RACMO experiments.

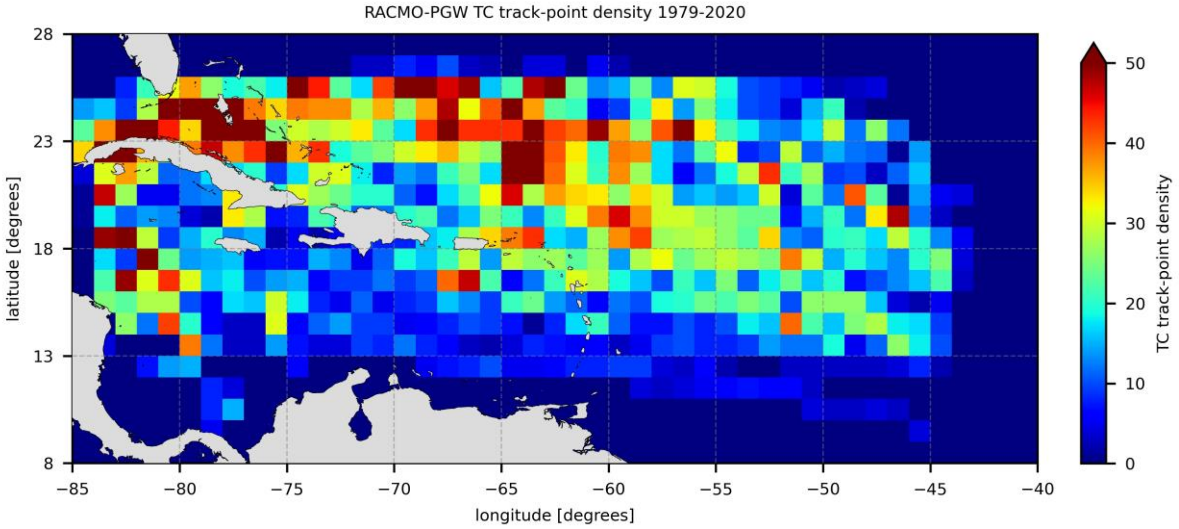

Supplementary Figure S7: Simulated TC track-point density (RACMO-PGW) within the RACMO model domain. Units are cumulative 1-hourly storm position frequency per 1° x 1° gridbox for the period 1979-2020.

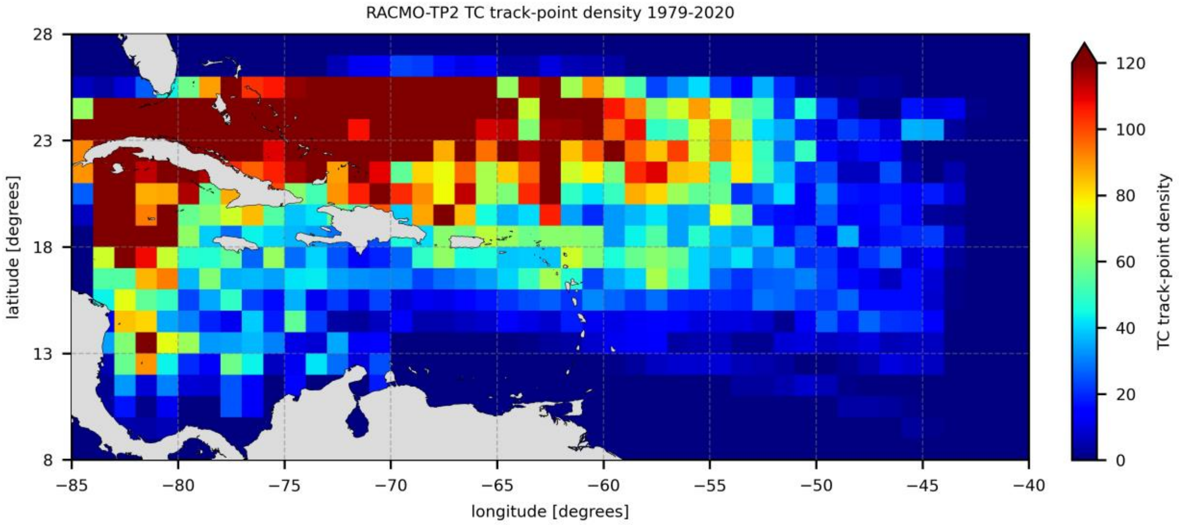

Supplementary Figure S8: Simulated TC track-point density (RACMO-TP2) within the RACMO model domain. Units are cumulative 1-hourly storm position frequency per 1° x 1° gridbox for the period 1979-2020.

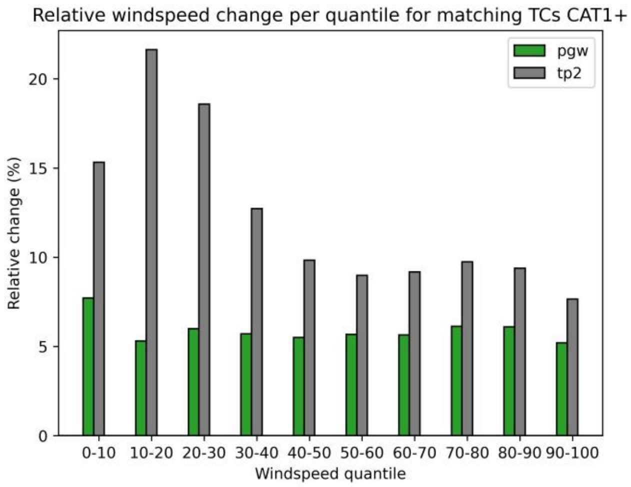

Supplementary Figure S9: bar plot showing the change in average hourly maximum wind speed per 10% quantile for TCs that reach at least category 1 on the Saffir-Simpson scale (wind speed exceeding 33 m/s).

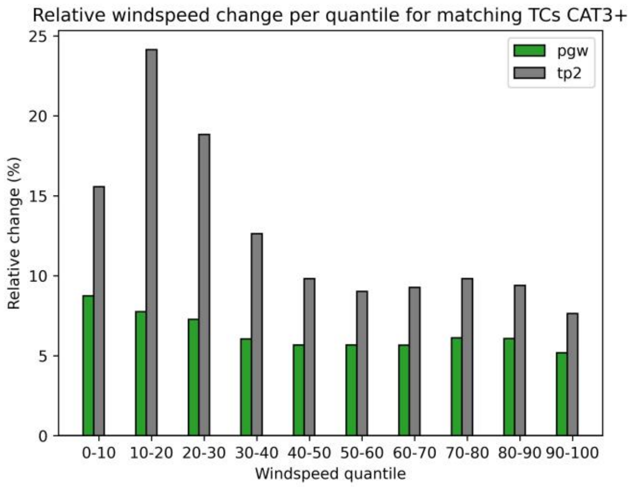

Supplementary Figure S10: bar plot showing the change in average hourly maximum wind speed per 10% quantile for TCs that reach at least category 3 on the Saffir-Simpson scale (wind speed exceeding 50 m/s).

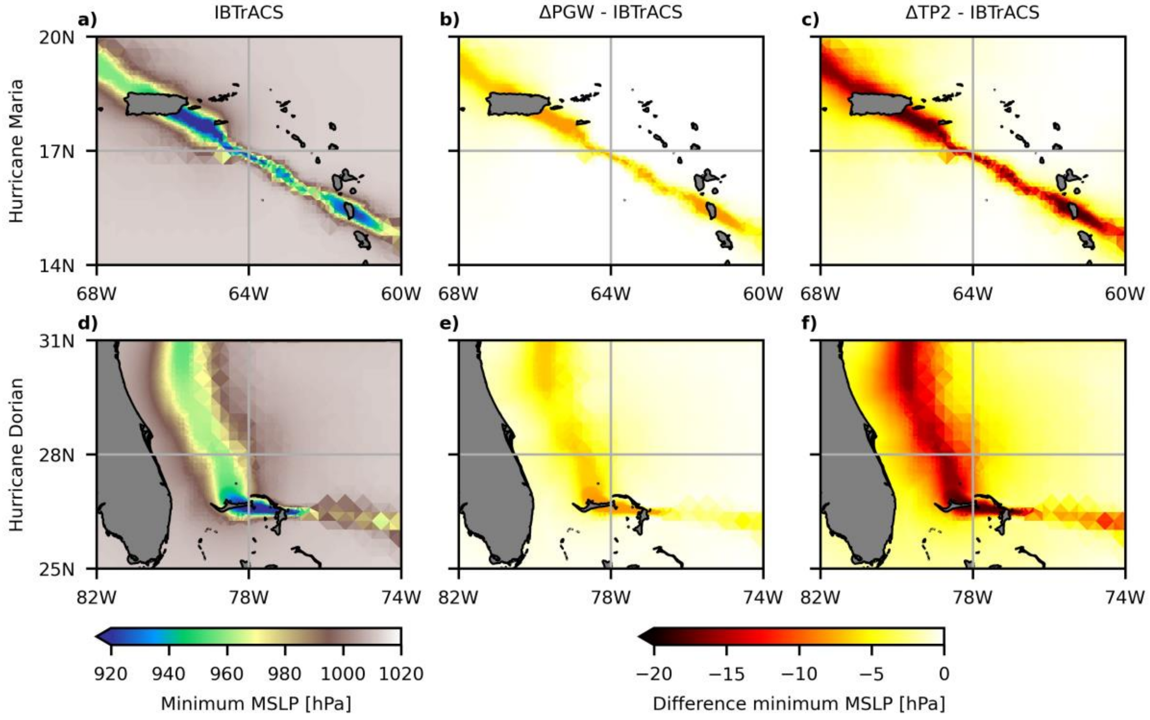

Supplementary Figure S11: a) minimum observed MSLP of Hurricane Maria (IBTrACS); b) difference in minimum MSLP using ΔPGW, calculated as ΔPGW minus IBTrACS; c) same as (b) but for ΔTP2; d-f) same as (a-c) but for Hurricane Dorian.

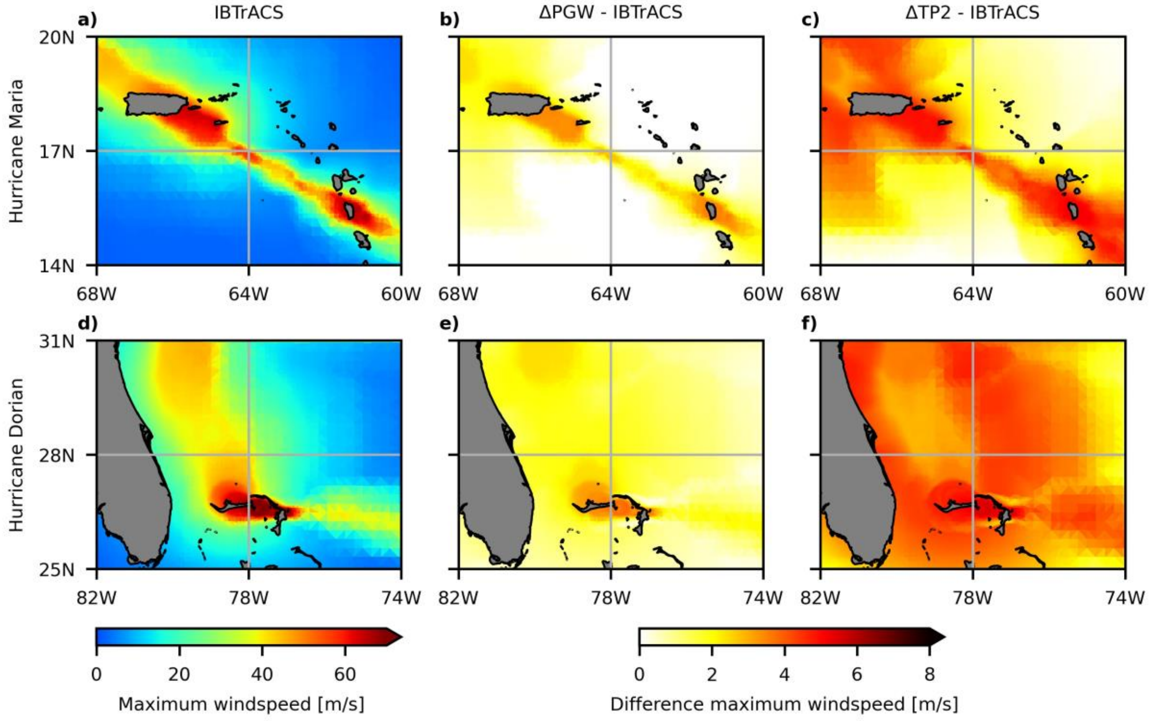

Supplementary Figure S12:. a) maximum observed wind speed of Hurricane Maria (IBTrACS); b) difference in maximum wind speed using ΔPGW, calculated as ΔPGW minus IBTrACS; c) same as (b) but for ΔTP2; d-f) same as (a-c) but for Hurricane Dorian.
